# Supplementary material for: The genome sequence of a caddisfly, Limnephilus auricula (Curtis, 1834)
Source: Wellcome Open Res. 2023 Dec 5;8:560. [Version 1] doi: 10.12688/wellcomeopenres.20383.1 (PMC11109698; doi:10.12688/wellcomeopenres.20383.1)
Supplement: Peer review history

# Reviewer report: Reviewer response for version 1

**DOI:** 10.21956/wellcomeopenres.22570.r76183

**Published:** 2024-05-15

**Authors:** Niclas Backström

**Affiliations:** 1 Department of Ecology and Genetics (IEG), Evolutionary Biology Program, Uppsala University, Uppsala, Sweden

The data note by McCulloch et al. describes the statistics of the genome assembly of a female Limnephilus auricula caddisfly. The results support a haploid karyotype consisting of 30 chromosomes, the Z-chromosome included. The analysis of scaffolds does not indicate the presence of a sex-limited chromosome (W) consistent with the established Z0 sex-chromosome system in trichoptera. The genome assembly is of high-quality with high N50 and N90 scores and >99.8% of the assembly assigned to chromosome level scaffolds. This resource will likely be useful for forthcoming population genomic studies within the genus / species and for comparative genomic studies in trichoptera in general (and for comparisons with other insect groups). The paper is well written and all results are presented in an accessible way.

 Minor comments:

 Maybe include a brief note on why the number of fragmented BUSCO genes is comparatively high (6.4%)? This results in that the proportion of complete BUSCOs is quite low (90.8%) for such an high-contiguity genome assembly.

 Maybe indicate in the Figure 5 legend that the Z-chromosome is the 4th largest (clearly visible from the lower signal in the contact map). The information is available in Table 2 of course, but it might be useful for readers to understand why the contact signal is reduced for that particular chromosome.

Are sufficient details of methods and materials provided to allow replication by others?

Yes

Is the rationale for creating the dataset(s) clearly described?

Yes

Are the datasets clearly presented in a useable and accessible format?

Yes

Are the protocols appropriate and is the work technically sound?

Yes

Reviewer Expertise:

Molecular evolution, population genomics

I confirm that I have read this submission and believe that I have an appropriate level of expertise to confirm that it is of an acceptable scientific standard.

# Reviewer report: Reviewer response for version 1

**DOI:** 10.21956/wellcomeopenres.22570.r73249

**Published:** 2024-02-22

**Authors:** Siegfried Roth

**Affiliations:** 1 Institute for Zoology, Universitat zu Koln, Cologne, North Rhine-Westphalia, Germany

This high-quality genome assembly of the caddisfly *Limnephilus auricula* will provide a useful tool for studying the Trichoptera, the sister taxon of the Lepidoptera. Moreover, the Trichoptera show unique adaptations to an aquatic life style. Hence comparative genomics with the basal lepidopteran lineages might uncover genomic signatures of these adaptations. The genome sequence spans 972.3 megabases and is scaffolded in 30 chromosomes. Assembly procedures and quality controls are well described and sufficiently referenced. The data are well presented. The work on the cytology of Trichoptera which support the presence of 30 haploid chromosomes in males should be referenced. In particular Kiauta and Lankhorst (1969) ^3^ have studied a species closely related to *Limnephilus auricula.*

Are sufficient details of methods and materials provided to allow replication by others?

Yes

Is the rationale for creating the dataset(s) clearly described?

Yes

Are the datasets clearly presented in a useable and accessible format?

Yes

Are the protocols appropriate and is the work technically sound?

Yes

Reviewer Expertise:

Evolutionary developmental biology of insects

I confirm that I have read this submission and believe that I have an appropriate level of expertise to confirm that it is of an acceptable scientific standard.

## References

[1] : Kinetic organization of metaphase I bivalents in spermatogenesis of Lepidoptera and Trichoptera species with small chromosome numbers. *Heredity* .1997;79(2) : 10.1038/hdy.1997.136 135-143 10.1038/hdy.1997.136

[2] : Large-scale comparative analysis of cytogenetic markers across Lepidoptera. *Sci Rep* .2021;11(1) : 10.1038/s41598-021-91665-7 12214 10.1038/s41598-021-91665-7 34108567 PMC8190105

[3] : The chromosomes of the caddis-fly, Glyphotaelius pellucidus (Retzius, 1783) (Trichoptera: Limnephilidae, Limnephilinae). *Genetica* .1969;40(1) : 10.1007/BF01787334 1-6 10.1007/BF01787334 5800688
